# Acceptability of “DIDE”, a mobile application designed at facilitating care adherence of patients with substance use disorder

**DOI:** 10.1186/s13722-024-00500-7

**Published:** 2024-10-15

**Authors:** Antoine Stocker, Nicolas Navarro, Laurent Schmitt, Marc Delagnes, Aurélie Doualle, Valérie Mallard, Flora Entajan, Karine Guivarc’h, Patricia Masse, Lilian Chaigneau, Baptiste Bonneau, Maryse Lapeyre-Mestre, Christophe Arbus, Antoine Yrondi, Juliette Salles

**Affiliations:** 1 Department of Child and Adolescent Psychiatry, Toulouse University Hospital, Toulouse, France; 2 CERPOP, Inserm, UPS, University of Toulouse 3, Toulouse, France; 3 CHU Toulouse, Service de Psychiatrie et Psychologie Médicale, Toulouse, F-31000 France; 4 Fédération Régionale de Recherche en Psychiatrie et santé mentale Occitanie, FERREPSY Occitanie, Toulouse, F-31000 France; 5 Unité MeDatAS-CIC 1436, Service de Pharmacologie Médicale et Clinique, CHU de Toulouse, Faculté de Médecine, Université Toulouse III, 37 allées Jules Guesde, Toulouse, 31000 France; 6 Toulouse NeuroImaging Center, ToNIC, University of Toulouse, Inserm, UPS, Toulouse, France; 7 Service de psychiatrie d’urgences, de crise et de liaison, CHU de Toulouse, Toulouse, France; 8 Infinity, Inserm UMR 1291, Equipe 5, Université Toulouse 3, Toulouse, France; 9 Institut des Handicaps Neurologiques, Psychiatriques et Sensoriels, CHU de Toulouse, Toulouse, France; 10 Service Universitaire de Psychiatrie de l’Enfant et de l’Adolescent, CHU de Toulouse, Hôpital Purpan, Place du Dr Baylac, TSA 40031, Toulouse cedex 9, 31059 France

**Keywords:** Addiction, eHealth, Mobile application, Treatment adherence and compliance, Therapeutic alliance

## Abstract

**Background:**

Attrition continues to be a major hurdle for addiction treatment. Through the prism of the attachment theory, this phenomenon can be understood as a manifestation of the patient’s insecure attachment style, needing a highly-responsive care delivery. We developed an electronic health mobile application, co-designed with patients, aimed at helping healthcare teams respond to their patients’ needs, and fostering adherence to care. This acceptability study evaluated patients everyday use of the application for eight weeks, assessing their satisfaction with the system, and its integration within professionals’ current practice in our center.

**Methods:**

This single-center, prospective study was conducted between January 2022 and December 2022. 24 adult patients with any type of addiction were included. They were granted access to the application for eight weeks, and were invited to complete the System Usability Scale questionnaire regarding their satisfaction with application’s usability at the end of the study. The application uses active self-reports, which are later discussed with the healthcare team, and foster both the working alliance and the decision-making process.

**Results:**

17 patients out of 24 reached the primary endpoint. On average, over the eight-weeks period, patients logged in the application 38.2 times, and sent 5.9 messages to the healthcare team. Interestingly, 64.3% of the user logins were recorded outside of our center’s working hours (either from 5 p.m. to 9 a.m., or during week-ends and bank holidays), and 70.8% of the patients logged into the application at least one time between 10 p.m. and 8 a.m. 18 patients completed the System Usability Scale questionnaire, which averaged a score of 81.8 out of 100. Healthcare professionals logged in the application’s messaging system 4.5 times a day on average.

**Conclusions:**

This preliminary study shows promising results, as patients engaged well with various components of the application. It was moreover possible for healthcare workers in our center to integrate this tool in their daily activities. More work is needed to better understand the various patients’ needs regarding the application, further strengthen their adherence to the intervention, and understand professionals’ motivations to use the application.

**Trial registration:**

ClinicalTrials.gov, Identifier: NCT04659954. Registered 09 December 2020, https://clinicaltrials.gov/study/NCT04659954.

**Supplementary Information:**

The online version contains supplementary material available at 10.1186/s13722-024-00500-7.

## Background

Substance Use Disorder (SUD) is a chronic disease frequently encountered in general or specialized practice, with a large prevalence rate: 46.3 million people aged 12 or older, representing 16.5% of the population, met the applicable Diagnostic and Statistical Manual of Mental Disorders 5th edition (DSM-5) criteria of SUD in 2023 in the USA [1]. Moreover, SUD represents a considerable socio-economic burden, evaluated by estimating disability-adjusted life years, which are the sum of years of life lost to premature mortality, and years of life lived with disability, and which were estimated in 2015 at 170.9 million for tobacco smoking, 85 million for alcohol and 27.8 million for illicit drugs [2]. In France, alcohol remains the most common preventable cause of death in the population before age 30, and the first cause of hospitalization. Moreover, within European countries, France ranks second in cannabis use, fourth in alcohol use and fifth in tobacco use among population aged 15 to 75 [3]. While cannabis use remains high, with 11.0% of French adults reporting last year use of cannabis, it remained stable between 2014 and 2017. Cocaine use however considerably increased in the country, with last year reported use going from 0.3% in 2000 to 1.6% in 2017, with a diffusion of based forms such as crack [4]. SUD treatment has thus to be considered a priority issue in public health policies. However, treatment adherence in SUD remains challenging, with a substantial proportion (ranging from 13 to 31%) of individuals not completing care programs [5, 6]. In addition, SUD treatment dropout is all the more a problem as it is a robust predictor of relapse [7–10].

Among different models, the attachment theory is an interesting approach to understand treatment dropout in SUD. Attachment development involves children developing trust in their parents’ support and protection during times of distress, allowing them to internalize early caregiving experiences into internal working models, with lasting effects on cognitive and socioemotional development throughout life [11, 12]. While a secure attachment fosters support seeking behaviors [13, 14], the lack of trust in the caregiver’s availability can lead to insecure attachment patterns, where individuals try to suppress the expression of negative emotions, leading to impairments in emotion regulation and increased impulsivity. Psychological factors associated with insecure styles of attachment, may therefore contribute to the development of SUD [15, 16] and to attrition from the treatment of these disorders [17–19]. However, attachment security remains open to change. Interventions aiming to help the patient shift from insecure to secure attachment style, by fostering trust in their relation with their caregivers, might therefore play an important role in SUD treatment. Such interventions focus on a better understanding of intense emotional reactions within the treatment setting, especially in relation with relatives, by clarifying and naming feelings; understanding of the immediate precipitant of emotional states within present circumstances; and appropriate, adequate, and constructive expression of feelings within the context of a relationship to the care team [20]. They are, however, not easily implemented in current practice, as they request a high availability and reactivity of the care team, including outside working hours.

Electronic health (eHealth) is burgeoning in the field of addiction, and is a promising tool for further improving the delivery of care to patients with SUD [21, 22]. From this evidence, we considered eHealth could help bridging the gap between the need of high availability and reactivity of care deliverability, and the limits of care resources. Indeed, patients with SUD more frequently present insecure attachment and thus difficulties to trust relationships and to seek help. Creating a therapeutic alliance is therefore more complicated with them, and requires more engagement from the care team. We thus designed an application named DIDE (the name of our local SUD treatment center, and a backronym from Digital Interaction for Detoxification Engagement) to help the care team build a therapeutic alliance by supporting more frequent contact with the patient while at the same time respecting their privacy. The core concept of the application is to serve as a companion of both the patient and the caregivers and to support attachment and the therapeutic relationship, by increasing the feeling of healthcare availability in order to support patient’s engagement in treatment. We thought that such a design could help strengthen the link between the patient and the caregivers and help fostering care adherence. Nevertheless, a first step is necessary to ensure that patients will adhere to the application concept, and make use of it in their everyday lives. Indeed, despite promises and some evidence of its efficacy, eHealth also faces strong attrition rates, partly related to participants not using technologies in the intended way [23]. Adherence to the eHealth interventions is thus crucial. Donkin et al. adapted World Health Organization’s definition of treatment adherence to define eHealth adherence as “the degree to which the user followed the program as it was designed” [24]. Based on this definition, the following elements are deemed necessary to determine adherence to eHealth technology: the ability to measure the usage behavior of individuals; an operationalization of intended use; and an empirical, theoretical, or rational justification of the intended use [25].

This study aims to document the adherence of patients with SUD to the DIDE application, over a two-months period of use, in addition to their usual care. The main objective is to measure the number of user logins in the application, while secondary objectives are the description of patients’ use of the different functionalities of the application, and the measurement of their satisfaction with the product.

## Methods

### Study design

This single-center prospective study aimed to evaluate the acceptability of the DIDE application (ClinicalTrials.gov Identifier: NCT04659954). Eligible patients were proposed to download and use the application during eight weeks, in addition to their usual treatment in the center (medical appointments, interviews with nurses, pharmacological treatment). The study was conducted in Toulouse University Hospital, France, between January 2022 and December 2022.

### Variables

Primary endpoint was first defined as the percentage of patients who logged into the application at least once every week. Some participants used the application on a more irregular basis over the study’s timeframe, as they felt the need to: this possibility was in line with existing data regarding mobile health use [26]. We thus modified our primary endpoint, to also include patients who logged into the application at least 10 times in four different weeks during the eight weeks of the study. User logins were recorded in real-time, and later separated in four groups for analysis: user logins during center’s working hours (Monday to Friday, 9 a.m. – 5 p.m.), evenings (every day, 5 p.m. – 10 p.m.) nights (every day, 10 p.m. – 9 a.m.) and week-end (Saturday, Sunday and bank holidays, 9 a.m. – 5 p.m.)

At the end of the study, participants were asked to complete the French-translated System Usability Scale (SUS) questionnaire, widely used in applications development and user experience design, in order to evaluate the usability of the application [27]. This questionnaire comprises 10 items, such as “I think that I would like to use this system frequently”, and aims to measure subjective impressions of a system’s usability, frequently defined as the combination of a system’s effectiveness, efficiency and the user’s satisfaction [28]. Each item is rated using a five-points Likert Scale, and the final score is comprised between 1 and 100.

### Participants

Participants were outpatients at our SUD treatment center, and were proposed to participate in the study during medical appointments. Eligibility criteria were as follows: presenting an active substance use addiction requiring weekly monitoring of consumption, possessing an Android smartphone or having access to an Android smartphone and having access to an internet connection, being able to read and understand French. Patients with cognitive or psychiatric disorders that may affect their ability to consent, or involved in another protocol involving a modification of the treatment for addiction were excluded from the study. Addiction diagnosis was determined after medical examination and based on DSM-5 criteria [29]. After they accepted to take part in the study, participants could download the application from Google’s Play Store, and had the application activated on their own smartphones. The application was operational during eight weeks from the installation, before its access was remotely revoked. A delay of two additional weeks was possible depending on the end of study visit date.

### Application design

The application was developed in collaboration with BotDesign, a partner start-up specialized in the development of eHealth solutions (https://www.botdesign.net). The application’s design is based on attachment theory and neurobiology of addiction, in order to help fostering the therapeutic alliance by proposing a system suited to answer the specific needs of patients with SUD. The application is centered around three core-concepts: ecological momentary assessment, messaging system with the healthcare team, and positive social reinforcement.

First, the choice of using ecological momentary assessment, in the form of self-report questionnaires later discussed with a caregiving team, was made for patients to more easily report their psychological state to the healthcare team, despite the possible fluctuation of symptoms [30], and the alteration of circadian rhythms [31]. We moreover considered self-report systems as a way to promote the declarative memory system, mediated by the hippocampus and hypothetically impaired by drugs of abuse to the benefit of habit memory, further reinforcing addiction [32]. Patients were prompted to complete questionnaires either through a conversation with a chatbot using multiple choices of answer, or by selecting a specific questionnaire. The first questionnaire, “Consumption diary”, acts as a self-report tool of the patient’s use of substance or addictive behavior, where patients can increment or decrement a counter through the day for each type of addiction specified within the application (e.g., cannabis, cocaine). The second questionnaire, entitled “Rhythm of life”, revolves around self-report of circadian rhythms of sleep and meals, which are altered in SUD [31]. A “Feeling” questionnaire was implemented, with the aim for patients to specify their overall well-being using a visual scale ranging from 0 to 10, in order to facilitate mentalization of their internal state. Patients also have the possibility, at any time, to report their craving using a dedicated questionnaire, with the option to specify the craving’s intensity, and to send a message to the caregiving team or receive information on available hotlines or forums for addiction. The questionnaires’ content is available in Additional File 1.

Second, the increased accessibility to caregivers was guaranteed by a messaging system, which the patient was systematically encouraged to use. Patients were informed that these messages were not to be considered as an emergency contact. However, they allowed them to leave a trace of their difficulties, so that the team could secondarily get in touch with them to propose appropriate support. Caregivers actively interacted with the patient during our service’s working hours, and showed their own interest in the intervention, which in turn was hypothesized to stimulate patients’ engagement in care. Data gathered within the app moreover served as decision-making support system for the team by providing supplementary clinical information.

Finally, the application provided positive social reinforcement, by using a considerate, valuing language and by displaying various messages to show concern to the user, congratulate or greet them. Combined with the possibility to review the application use with the caregiving team, these elements aimed to renew the patient’s interest in social interactions, hindered by SUD induced hypohedonic state and impairment of social skills [33].

Healthcare professionals in the center were granted a distinct access to the application system, using a dedicated desktop interface, that allowed them to use the messaging system with the patients, and to monitor data from individual questionnaires. The system was presented as a tool to help fostering therapeutic alliance with SUD patients, and was integrated in the center’s daily functioning. The team took additional time during their weekly clinical meetings to review completion of questionnaires, which helped decision-making processes regarding patients’ treatment, and helped them to prioritize patients they felt were facing more difficulties.

In order to maximize patients’ engagement, we used codesign techniques during the application’s development. Prior to the start of this study, patients with SUD were received in the center where the application would later be deployed. Voluntary patients were asked general questions about smartphone possession and usage, and their interest in health applications, before being presented with a prototype of the application, and asked to accomplish specific tasks. Additional feedback on the prototype was gathered during two supplementary sessions, during which one researcher (AS), as well as user experience designers from our partner start-up, asked the users their opinion and ideas on the application, their feedback on the questionnaires and the overall wording of the application’s content, and observed their interaction with the interface. This feedback was implemented in subsequent versions of the application. The application was not modified by users’ feedback during the course of study.

### Ethics

This research received a favorable evaluation from the French Committee for the Protection of Persons Sud Méditerranée I (Marseille, France), and was registered under the internal reference number 20 93.

### Statistical analysis

A distinction was made between categorical (e.g., professional status, housing) and quantitative variables. Percentages were calculated for each categorical variable possible values, while for quantitative variables, a set of descriptive statistical measures, including the mean with the standard deviation, was studied. Additionally, boxplot graphs were used to observe the distribution of quantitative variables.

Confidence intervals with a 95% confidence level were constructed to provide a plausible range of values for the percentages of categorical variables and also for the means of quantitative variables. Due to the small sample size and non-normality of the data, assessed using the Shapiro-Wilk test, specific tests were used to calculate these intervals. The Bootstrap percentile method was employed for quantitative variables, while the Clopper-Pearson Mid P method was used when two modalities were present for categorical variables, and the multinomial method when three or more modalities were present. All analyses were conducted using R Studio - version 4.2.1.

## Results

### Population description

**Fig. 1:**
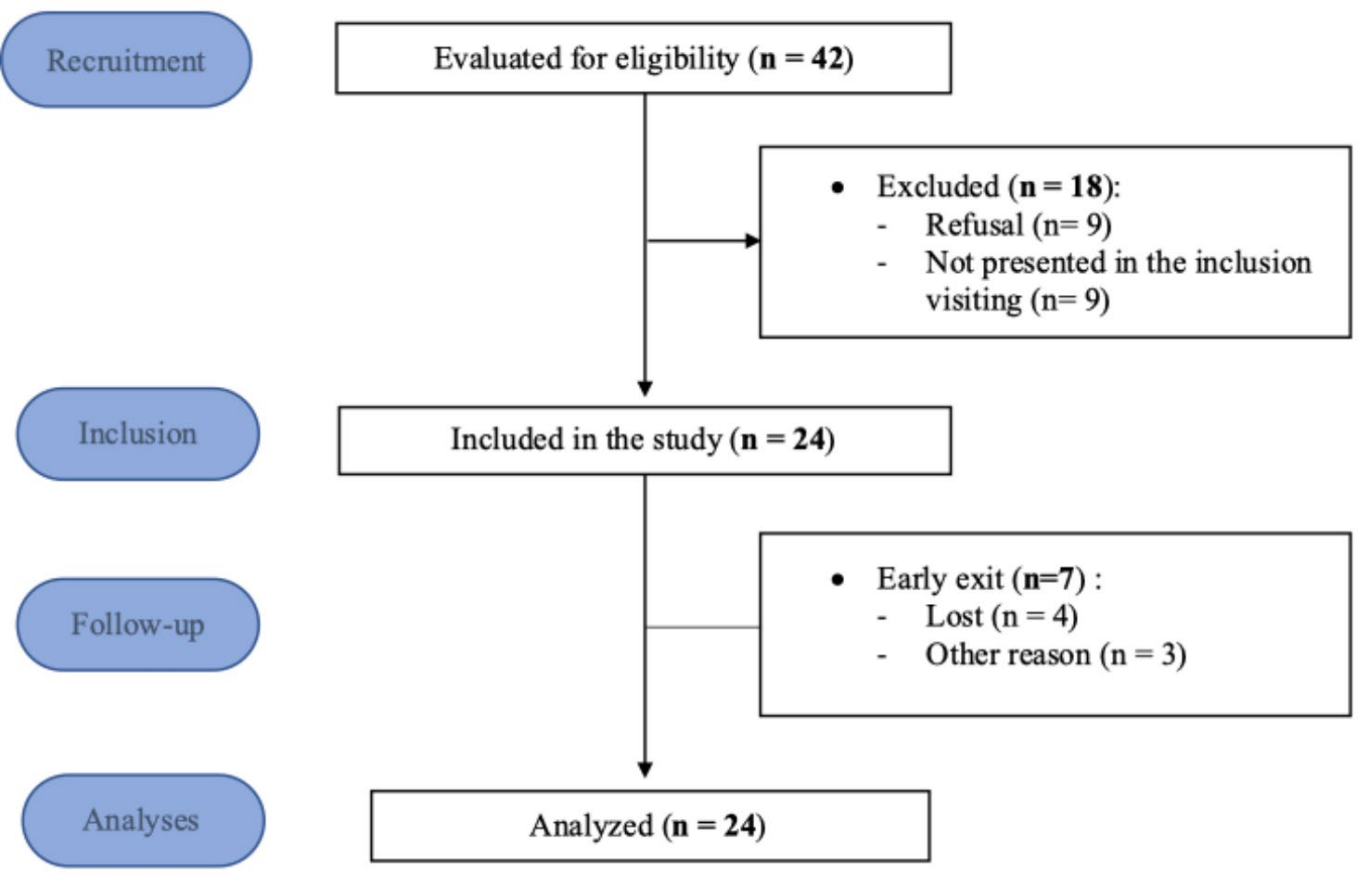
Flow chart of the study

Forty-two patients were deemed eligible to the study during the recruitment period. From these, 24 (57%) were included in the study. The flow chart (Fig. 1) describes the inclusion and analyze process.

Patients’ mean age was 30 years. 58% of the participants were women. Most participants were students (58%), and all but one had stable housing. Half of them were single (54%) and the majority had no children (92%). Most of the participant had a psychiatric diagnosis associated with the addiction (75%). Anxiety, depression, and suicidal behavior represented near 75% of the diagnoses. 70.9% of the patients had ongoing pharmacological treatment, either for a psychiatric indication (54.2%), or both psychiatric and SUD indications. Interestingly, no included patient had a pharmacological treatment for SUD alone. A complete population description is presented in Table 1. Patients mainly consulted for cannabis addiction (54%), and most had an addiction classified as severe according to the DSM-5 criteria (88%). 80% presented a co-addiction, that consisted in alcohol consumption in 42% of the cases. The complete description of our sample’s addiction profile is reported in Table 2.

**Table 1:** Description of our population characteristics

	Patients who meet primary endpoint (*N* = 17)	Patients who do not meet primary endpoint (*N* = 7)	Total sample (*N* = 24)
**Age, mean (SD)**	32.7 (13.3)	25.4 (3.4)	30.6 (11.8)
**Gender**			
Women	13 (76.5%)	1 (14.3%)	14 (58.3%)
Men	4 (23.5%)	6 (85.7%)	10 (41.7%)
**Professional status**			
Employed	4 (23.5%)	1 (14.3%)	5 (20.8%)
Student	7 (41.2%)	4 (57.1%)	11 (57.9%)
Unemployed	5 (29.4%)	2 (28.6%)	7 (36.8%)
Invalidity	1 (5.9%)	0 (0%)	1 (5.3%)
**Housing**			
Stable	16 (94.1%)	7 (100%)	23 (95.8%)
Other	1 (5.9%)	0 (0%)	1 (4.2%)
**Relationship status**			
In a relationship	9 (52.9%)	2 (28.6%)	11 (45.8%)
Single	8 (47.0%)	5 (71.4%)	13 (54.2%)
**Children**			
Yes	2 (11.8%)	0 (0%)	2 (8.3%)
No	15 (88.2%)	7 (100%)	22 (91.7%)
**Previous psychiatric history**			
Yes	13 (76.5%)	5 (71.4%)	18 (75.0%)
No	4 (23.5%)	2 (28.6%)	6 (25.0%)
Number of past diagnoses per patient, mean (SD)	2.6 (2.0)	1.6 (1.4)	2.3 (1.9)
**Type of previous psychiatric diagnoses**			
Anxiety	10 (58.8%)	3 (42.9%)	13 (54.2%)
Suicidal behavior (suicide attempts, suicidal ideation, self-injury)	11 (64.7%)	2 (28.6%)	13 (54.2%)
Depression	8 (47.1%)	3 (42.9%)	11 (45.8%)
Eating disorders	5 (29.4%)	1 (14.3%)	6 (25.0%)
Sleep disorders	2 (11.8%)	1 (14.3%)	3 (12.5%)
Substance withdrawal symptoms	2 (11.8%)	0 (0%)	2 (8.3%)
Schizophrenia	1 (5.9%)	0 (0%)	1 (4.2%)
ADHD	0 (0%)	1 (14.3%)	1 (4.2%)
Borderline personality disorder	3 (17.6%)	0 (0%)	3 (12.5%)
Substance-induced psychotic disorder	1 (5.9%)	0 (0%)	1 (4.2%)
Bipolar disorder	1 (5.9%)	0 (0%)	1 (4.2%)
**Ongoing concurrent psychiatric or addiction pharmacological treatment**			
Yes (psychiatric treatment only)	10 (58.8%)	2 (28.6%)	13 (54.2%)
Yes (addiction treatment only)	0 (0%)	0 (0%)	0 (0%)
Yes (both)	3 (17.6%)	2 (28.6%)	4 (16.7%)
No	4 (23.5%)	3 (42.9%)	7 (29.2%)
**Type of ongoing pharmacological treatment** ^a^			
Antidepressant	9 (52.9%)	3 (42.9%)	12 (50.0%)
Anxiolytic	9 (52.9%)	4 (57.1%)	15 (62.5%%)
Neuroleptic	8 (47.1%)	0	8 (33.3%)
Substitutive treatment	3 (17.6%)	1 (14.3%)	4 (16.7%)

Table values are provided as n (%), unless otherwise specified

^a^ Values presented represent the number of patients receiving at least one molecule of the treatment class

Additional file 1.docx : Application questionnaires

**Table 2:** Addiction profile within study participants

	*N* = 24 (100%)
**Main addiction**	
Cannabis	13 (54.2%)
Alcohol	5 (20.8%)
Cocaine	3 (12.5%)
Opioids	2 (8.3%)
Other	2 (8.4%)
**Addiction severity (DSM-5)**	
Severe	21 (87.5%)
Moderate	3 (12.5%)
**Co-addiction**	
Yes	19 (79.2%)
No	2 (8.3%)
Missing data	3 (12.5%)
**Type of Co-addictions** (*n* = 15)	
Alcohol	8 (42.1%)
Cocaine	5 (26.3%)
Cannabis	1 (5.3%)
Other	1 (5.3%)

### Use of the application

Among the 24 patients enrolled in the study, 17 (70.8%) logged into the application at least once per week during the first eight weeks, or more than 10 times over at least four different weeks. Five participants (20.8%) did log into the application at least once per week for the entirety of the study. The patient with the highest number of user logins registered 270 logins, while the one with the fewest number registered two. Most of the user logins were recorded in the first week after the application’s activation (*n* = 273, 30.2%), with a sudden drop observed in the second week (*n* = 139, 15.4%) and a steady decline over the following weeks (*n* = 80, 8.8% during the final week). However, when considering the number of participants logging into the application each week, this decrease was less important: all participants logged at least once into the application during the first week, 18 (75%) during the second week and 10 (41.7%) during the final week.

Of the 904 user logins recorded, 725 (80.2%) occurred between the hours of 8 a.m. – 10 p.m., while 179 (19.8%) occurred between the hours of 10 p.m. – 8 a.m. Additionally, it should be noted that all patients logged in at least once between 8 a.m. and 10 p.m. However, only 17 patients (70.8%), logged in between 10 p.m. and 8 a.m.

**Fig. 2:**
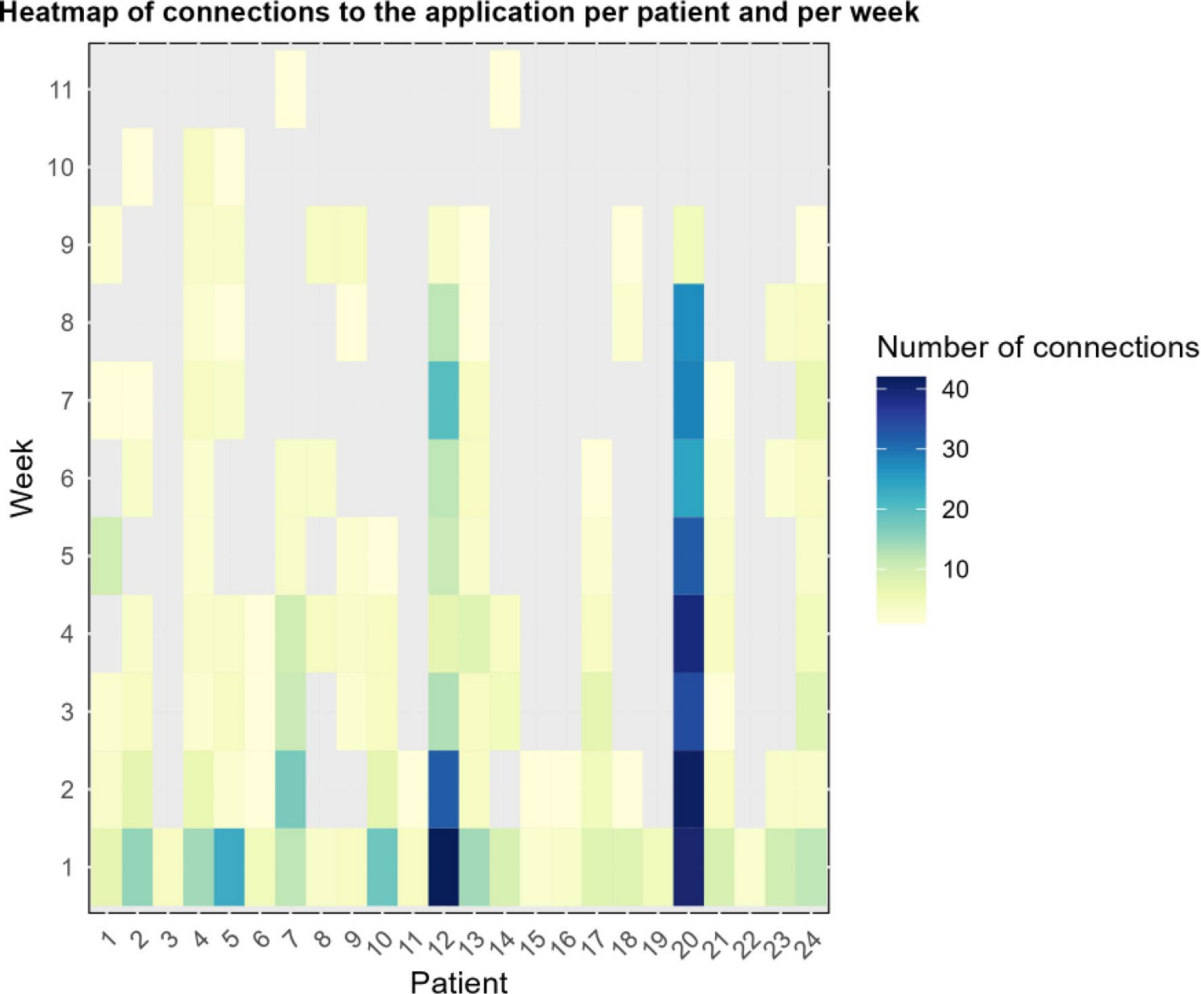
Number of user logins for each patient per week over the period of application activation. Please note, that data from weeks 10 and 11 correspond to login attempts after the end of the protocol, when patients’ access to the application was revoked

Interestingly, out of the 904 total user logins, only 323 (35.7%) took place during our healthcare service’s working hours (from 9 a.m. to 5 p.m.). The remaining 581 (64.3%) recorded logins correspond to 280 logins in the evening (5 p.m. – 10 p.m.), 197 logins at night (10 p.m. – 9 a.m.) and 104 logins during week-ends and bank holidays (9 a.m. – 5 p.m.) The number of logins per patient over the protocol period is presented in Fig. 2.

The seven patients who did not meet primary endpoint only logged into the application 31 times (3.4%). The majority (61.3%) of these logins were recorded during our service’s working hours. More than half of these logins (54.8%) were done on the first day of access to the application.

Each individual made an average of 18.9 consumption reports *via* the app over the eight weeks. The maximum number of reports made by a patient was 64, while the minimum was zero. Among the 20 patients who responded at least once to the “Consumption diary” questionnaire, the questionnaire was filled 22.7 times on average. The patient with the highest number of responses made 64, while the patient with the fewest made one. The “Rhythm of life” questionnaire was filled on average 14.5 times, the maximum number of responses for one patient being 54, and the minimum one. The “Feeling” questionnaire was filled 18.1 times on average. The highest-responding patient completed the form 57 times, whereas the lowest-responding only did once. Finally, the craving questionnaire averaged 13.5 responses. The patient with the highest number of responses made 41, while the patient with the fewest made one. The application did not send reminders to complete questionnaires to the patients. However, the application’s design let the possibility for participants to complete questionnaires multiple times a day. This was especially relevant for context-dependent questionnaires such as “Craving” and “Consumption diary”.

The average number of messages sent to the healthcare team via the application over the eight weeks was 5.9 per patient. The patient who sent the fewest messages sent zero, while the one who sent the most sent 26. Average app logins per patient over the eight weeks was 38.2.

### SUS score

Among the 24 patients in the study, 18 (75%) completed the SUS questionnaire. The six patients who did not were all among the seven patients who did not complete the study according to the protocol, three of which did not meet the primary endpoint. The average of the scores obtained is 81.1 out of 100. The lowest score attributed to the application was 35, and the highest 97.5. Mean score of the 14 patients who met primary endpoint was 80.36, and 83.75 in patients who did not meet primary endpoint. While the score of the SUS questionnaire is subject to interpretation, its mean value of 81.1 indicates an overall satisfying user experience, which can however be further perfected [34].

### Healthcare team contacts to the patient and application use

Professionals used the application on a day-to-day basis. 2032 professional logins were recorded over the course of the study. The healthcare team made an average of 2.9 contacts with each patient, using the application’s internal messaging system. The minimum number of contacts made with a patient by the healthcare team was one, while the maximum was 19. On average, healthcare professionals logged in the messaging system 4.5 times a day over the eight weeks.

### Application attrition

**Fig. 3:**
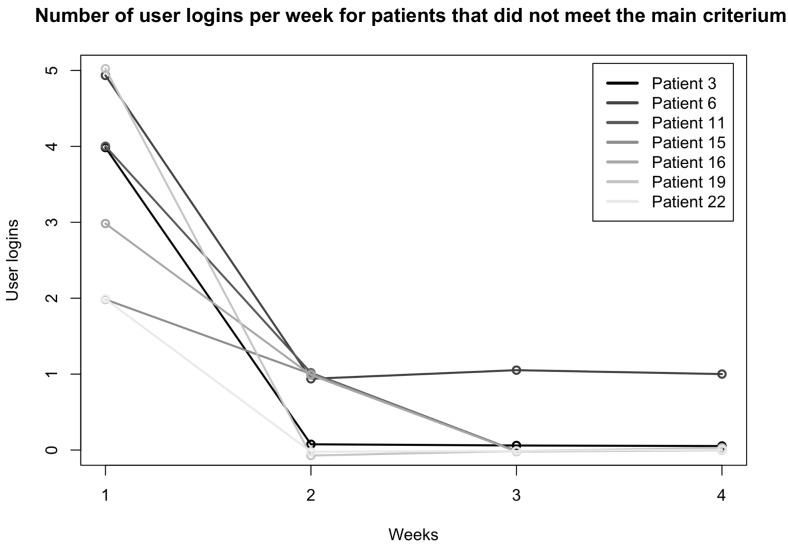
Number of user logins per week for patients that did not meet the primary endpoint. For example, patient 11 logged four times into the application in the first week, then only one time in the second week. Patient 6 logged five times into the application the first week, then one time at weeks 2, 3 and 4

We noted that among the seven patients who did not meet the modified primary endpoint, all connected in the first week, only 4 of them connected in the second week, and only one patient (patient 6), connected in weeks 3 and 4. The Fig. 3 presents the evolution of the user logins for each these seven patients.

## Discussion

The application presents a good acceptability rate from the patients, of which 70.8% met our primary endpoint. Over the course of the study, patients engaged less regularly and less frequently with the app. However, more than a third of our sample logged into the application during the last week of their participation. Other applications in the field of SUD treatment presented higher long-term use rates: the A-CHESS and mWSPARCIE applications for patients with Alcohol Use Disorder respectively had 78.2% and 61% of regular users at the end of their four and six months study periods [35, 36]. The SMART Track application, which was provided in addition to SUD mutual recovery groups, was used by 58% of participants after five weeks in an eight-week study, albeit with intermittent use [37].

However, these values are highly dependent on the design and purpose of each application, and on the healthcare context in which they are delivered. SMART Track and mWSPARCIE both used push notifications to foster users’ engagement, while access to A-CHESS and mWSPARCIE was provided during residential or inpatient treatment, respectively. In the absence of a standard way to measure adherence to eHealth interventions, Jakob et al. proposed the evaluation of an adherence score (calculated as a ratio of intended use, in our case weekly, to actual use). Our score of 56.7% is in the upper tier of SUD-designed interventions, which interestingly had the lowest mean adherence score of the included noncommunicable diseases (mean score 46.1%, SD 33.0%) [38]. Yet, interventions included in this systematic review recruited overall older participants (mean age 40.8 years, SD 9.6), with a slightly smaller proportion of women (mean 49.3%, SD 14.1%). Our protocol lasted slightly longer than the median intervention duration of included studies (56 days, interquartile range 90.5–17.6). Most of the included studies moreover focused on tobacco and alcohol use, while we proposed our application to an outpatient population predominantly made up of cannabis users with frequent co-addictions [38]. While we made extensive use of feedback systems, the only identified positive factor on adherence in Jakob et al. systematic review for multi-domain SUD application [38] which may have contributed to the higher adherence seen within our study, this factor was also identified in a study of an application with a different target population of non-treatment seeking college students with concurrent heavy-episodic drinking and smoking problems [39].

The majority of the user logins were made outside of the center’s working hours, which may indicate that the application helped patients to overcome the limits of care availability. Similarly, most of user logins into the SMART Track app were made between 6 p.m. and 9 p.m. (accounting for 23.1% of overall logins) [37].

Healthcare professionals used the application on a daily basis to engage with their patients, as part of their daily activities in the center. In comparison to a mobile app dedicated to Opiod Use Disorder (HOPE app), professionals in our team seemed to send less messages on average (2.9 contacts versus 8.9 in the HOPE app), despite similar number of messages from participants to their team in both applications (mean of 5.9 messages versus 7.5 messages in the HOPE app) [40]. This difference can be explained by a very positive experience of care providers with the HOPE application’s messaging system (which we did not evaluate for our application in this study), and to a lesser extent, by the fact that researchers could also send messages in the HOPE application.

Our study faced a 29.2% attrition rate. While important, it remains in line with the 30.4% average attrition rate found in studies of SUD in-person psychosocial treatment [7]. Moreover, among the seven patients who did not meet our primary endpoint, three did so because they dropped out of ambulatory care due to a worsening of their SUD or comorbid psychiatric condition. As more than half of these patients’ logins to the application were recorded during or shortly after the appointment during which access to the application was granted, it is possible that these patients’ interest in this system was low from the very beginning of the study.

More than half of the patients (58.3%) completed two or more questionnaires each day they used the app, and only one participant, who did not meet our endpoint, never filled any questionnaire. The “Feeling” questionnaire and the “Consumption diary” seemed to gain more traction from users. Systems related to self-observation of craving and consumptions were the most accessed by users in the Step Away and mWSPARCIE mobile applications for Alcohol Use Disorder [36, 41]. The lesser use of the “Craving” questionnaire in our study might point out a better clinical state, an underreporting of those moments, or an inadequate presentation of this reporting system.

Our questionnaire on rhythms of life was also less accessed by users. This can indicate that patients might consider questions on this topic irrelevant to their treatment, or too intrusive. A recent cross-sectional study on patients’ interest in SUD applications showed that the majority of participants reported privacy as a primary concern [42]. However, most participants felt comfortable with the idea of “coaching for healthy living”, to which our questionnaire on rhythm of life relate. Moreover, Jakob et al. review indeed pinpointed the negative effects of a lack of eHealth and health literacy on adherence [38]. It is thus possible to think that patients would make a better use of this functionality with clearer explanations from the caregiving team.

Interestingly, the five patients who completed the most questionnaires each day (mean values ranging from 3.3 to 4) made a homogeneous use of every questionnaire. This is in line with findings regarding the uttermost importance of acceptability of eHealth interventions in SUD, where better engagement usually lead to better outcomes [43].

Patients overall rated their user experience as satisfying with a mean SUS score of 81.1, in line with the HOPE app’s mean score of 86.9 [40], and the Step Away mean score of 71.9 after 6 months of use [41]. They moreover provided nuanced feedback on their experience with the application during SUS questionnaire completion. Three participants expressed a lack of interest towards the application. Three other participants, on the contrary, felt the application was helpful and interesting, and two wished to keep using the application after our study’s end. Feedback on the messaging system, and on the healthcare team availability, was positive. Patients often felt “Feeling” and “Rhythm of life” questionnaires were too simplistic. “Craving” and “Consumption diary” questionnaires were overall perceived as useful, however, two patients felt too strong craving to fill the questionnaires, and one expressed self-stigma when recording her consumptions in the diary. Participants regretted the lack of push notifications and reminders to complete questionnaires. Patients moreover provided feedback on functionalities they wish to use within the application, such as a notepad, more context-dependent and personalized notifications, or gamification elements. Some of those elements were already considered by our team, most notably gamification processes, which showed a positive effect on user’s engagement in eHealth [44]. Although these elements are heterogeneous in their effect and need to be tailored to target populations to remain relevant [45], they are more and more implemented in eHealth interventions for SUD [46].

Finally, as the Lancet Commission on the Future of Psychiatry pointed out, both clinicians and patients’ commitment to an eHealth dispositive is of prime importance [47]. In this regard, we strived to design an application that could be seamlessly integrated as part of the care process in our center, and that would act as a support of healthcare provision and therapeutic alliance. The application was proposed to our caregiving team as an additional tool to contact patients, discuss their needs and adjust proposed care. Strikingly, the impact of human support when using eHealth devices is not clearly demonstrated across various domains of mental health [48], as well as the correlation between level of personal support and adherence to SUD eHealth applications, which was non-significant [38]. However, our choice to integrate human support in the application was not primarily designed to ensure patient adherence to the eHealth intervention, but to support patients’ adherence to traditional healthcare. As this was a pilot study, we did not assess patients’ adherence to care, but will conduct a larger randomized controlled trial to assess the effect of an upgraded version of our application on therapeutic alliance.

Our study is mostly limited by its small sample size and single-center design, leading to an important sample bias. Indeed, most of the patients we recruited were students (57.9%). Included patients moreover presented a high rate of cannabis use disorder as well as frequent co-addictions. Such profiles are different from the usual treatment-seeking population in French outpatient clinics for care, support and prevention in addictology [3]. The characteristics of our sample, as well as the French context of SUD epidemiology and specificities in healthcare provision therefore limit the generalizability of our results. The study’s short duration prevented us from ensuring that patients would use the application over a longer term, which is problematic considering that previous studies on eHealth both in SUD [43] and in the general population [49] reported lesser adherence to health applications over longer periods of time. We nonetheless strived to develop our application as close as possible to the patients’ needs, by including both evidence-based knowledge regarding SUD and by involving patients with SUD at various points of the app development, including early stages. Thus, our choices of design (i.e., involvement of users, seamless integration of the application in real-life clinical practice and care, ethical consideration on data and compliance with the European General Data Protection Regulation standards) are in line with findings of the literature as well as recent expert consensus on eHealth implementation and evaluation [50]. Acceptability of the system for health professional was only assessed through measures of their logins in the application and use of the messaging system. While our team managed to integrate this application in their daily activities, a proper investigation of their experience in using eHealth intervention with patients is lacking. Further work on this matter is needed to ensure eHealth adoption in professionals, using qualitative research methodologies for example. Additional data are also required to evaluate our application’s real world use, for eHealth applications are known to show better adherence rates when they are distributed within a scientific study rather than when they are accessible publicly [38].

## Conclusions

Despite its limitations, this single-center acceptability study of a novel eHealth intervention aimed at patients with SUD shows promising results. Patients who used the application seemed to engage well with its various components, and healthcare professionals were able to integrate this application in their daily activities. This preliminary work moreover allowed our team to gather more feedback on the application, and to prioritize useful functions that need to be implemented in subsequent versions of the system (e.g., a more complete self-reporting questionnaire of patient’s emotions to foster mentalization of their internal state, or to-do lists shared with the caregivers to work on motivation). We used a scientific rationale on SUD as well as codesign techniques to maximize the efficiency of our dispositive, whose effect on patient’s engagement to care and working alliance shall be studied in an upcoming, larger-scale comparative trial.

## Electronic supplementary material

Below is the link to the electronic supplementary material.

Supplementary Material 1

## Data Availability

Data and materials are available upon request, by contacting the corresponding author.

## Abbreviations

DSM-5: Diagnostic and Statistical Manual of Mental Disorders, 5th edition
eHealth: Electronic health
SUD: Substance Use Disorder
SUS: System Usability Scale

## References

[1] Administration (SAMHSA) SA. and MHS. HHS.gov. 2023 [cited 2023 Jul 26]. SAMHSA Announces National Survey on Drug Use and Health (NSDUH) Results Detailing Mental Illness and Substance Use Levels in 2021. https://www.hhs.gov/about/news/2023/01/04/samhsa-announces-national-survey-drug-use-health-results-detailing-mental-illness-substance-use-levels-2021.html

[2] Peacock A, Leung J, Larney S, Colledge S, Hickman M, Rehm J, et al. Global statistics on alcohol, tobacco and illicit drug use: 2017 status report. Addict Abingdon Engl. 2018;113(10):1905–26. 10.1111/add.14234 29749059

[3] Observatoire français des drogues et des tendances addictives. Drugs and Addictions Key Daya [Internet]. 2022 [cited 2024 Apr 25]. https://en.ofdt.fr/BDD/publications/docs/DACC_2022_EN.pdf

[4] Le Nézet O, Martinez M, Gérome C, Gandilhon M, Janssen E, Drugs. 2021 National Report (2020 data) to the EMCDDA by the Reitox National Focal Point France. OFDT; 2021.

[5] Daughters SB, Lejuez CW, Bornovalova MA, Kahler CW, Strong DR, Brown RA. Distress tolerance as a predictor of early treatment dropout in a residential substance abuse treatment facility. J Abnorm Psychol. 2005;114(4):729–34. 16351393 10.1037/0021-843X.114.4.729

[6] Daughters SB, Stipelman BA, Sargeant MN, Schuster R, Bornovalova MA, Lejuez CW. The interactive effects of Antisocial personality disorder and court-mandated status on substance abuse treatment dropout. J Subst Abuse Treat. 2008;34(2):157–64. 17869050 10.1016/j.jsat.2007.02.007 PMC3586262

[7] Lappan SN, Brown AW, Hendricks PS. Dropout rates of in-person psychosocial substance use disorder treatments: a systematic review and meta-analysis. Addiction. 2020;115(2):201–17. 31454123 10.1111/add.14793

[8] Carroll KM. Enhancing retention in clinical trials of psychosocial treatments: practical strategies. NIDA Res Monogr. 1997;165:4–24. 9243544

[9] Ciraulo DA, Piechniczek-Buczek J, Iscan EN. Outcome predictors in substance use disorders. Psychiatr Clin North Am. 2003;26(2):381–409. 12778840 10.1016/s0193-953x(02)00106-5

[10] Gainey RR, Wells EA, Hawkins JD, Catalano RF. Predicting treatment retention among cocaine users. Int J Addict. 1993;28(6):487–505. 8486433 10.3109/10826089309039643

[11] Fonagy P. Attachment, trauma, and psychoanalysis: where psychoanalysis meets neuroscience. Early Development and its disturbances. Routledge; 2010.

[12] Bowlby J. The making and breaking of affectional bonds. I. Aetiology and psychopathology in the light of attachment theory. An expanded version of the Fiftieth Maudsley lecture, delivered before the Royal College of Psychiatrists, 19 November 1976. Br J Psychiatry J Ment Sci. 1977;130:201–10. 10.1192/bjp.130.3.201 843768

[13] Cassidy J. The nature of the child’s ties. Handbook of attachment: theory, research, and clinical applications. 2nd ed. New York, NY, US: The Guilford Press; 2008. pp. 3–22.

[14] Dujardin A, Santens T, Braet C, De Raedt R, Vos P, Maes B, et al. Middle Childhood support-seeking behavior during stress: Links with Self-reported attachment and future depressive symptoms. Child Dev. 2016;87(1):326–40. 26822450 10.1111/cdev.12491

[15] Hiebler-Ragger M, Unterrainer HF. The Role of Attachment in Poly-Drug Use Disorder: An Overview of the Literature, Recent Findings and Clinical Implications. Front Psychiatry [Internet]. 2019 Aug 27 [cited 2020 May 2];10. https://www.ncbi.nlm.nih.gov/pmc/articles/PMC6720034/10.3389/fpsyt.2019.00579 PMC6720034 31507461

[16] Schindler A, Attachment. and Substance Use Disorders—Theoretical Models, Empirical Evidence, and Implications for Treatment. Front Psychiatry [Internet]. 2019 [cited 2023 Aug 4];10. https://www.frontiersin.org/articles/10.3389/fpsyt.2019.00727 10.3389/fpsyt.2019.00727 PMC6803532 31681039

[17] Fowler JC, Groat M, Ulanday M. Attachment style and treatment completion among Psychiatric inpatients with Substance Use disorders. Am J Addict. 2013;22(1):14–7. 23398221 10.1111/j.1521-0391.2013.00318.x

[18] Caspers KM, Yucuis R, Troutman B, Spinks R. Attachment as an organizer of behavior: implications for substance abuse problems and willingness to seek treatment. Subst Abuse Treat Prev Policy. 2006;1(1):32. 17081298 10.1186/1747-597X-1-32 PMC1635415

[19] Rübig LL, Fuchshuber J, Köldorfer P, Rinner A, Fink A, Unterrainer HF. Attachment and Therapeutic Alliance in Substance Use Disorders: Initial Findings for Treatment in the Therapeutic Community. Front Psychiatry [Internet]. 2021 [cited 2023 Aug 4];12. https://www.frontiersin.org/articles/10.3389/fpsyt.2021.730876 10.3389/fpsyt.2021.730876 PMC8631432 34858223

[20] Bateman AW, Fonagy P. The development of an attachment-based treatment program for borderline personality disorder. Bull Menninger Clin. 2003;67(3):187–211. 14621062 10.1521/bumc.67.3.187.23439

[21] Ferreri F, Bourla A, Mouchabac S, Karila L. e-Addictology: an overview of New technologies for assessing and intervening in addictive behaviors. Front Psychiatry. 2018;9:51. 29545756 10.3389/fpsyt.2018.00051 PMC5837980

[22] Bahadoor R, Alexandre JM, Fournet L, Gellé T, Serre F, Auriacombe M. Inventory and Analysis of Controlled Trials of Mobile Phone Applications Targeting Substance Use Disorders: A Systematic Review. Front Psychiatry [Internet]. 2021 [cited 2023 Aug 4];12. https://www.frontiersin.org/articles/10.3389/fpsyt.2021.622394 10.3389/fpsyt.2021.622394 PMC7937918 33692708

[23] Kohl LFM, Crutzen R, de Vries NK. Online prevention aimed at lifestyle behaviors: a systematic review of reviews. J Med Internet Res. 2013;15(7):e146. 23859884 10.2196/jmir.2665 PMC3714003

[24] Donkin L, Christensen H, Naismith SL, Neal B, Hickie IB, Glozier N. A systematic review of the impact of adherence on the effectiveness of e-therapies. J Med Internet Res. 2011;13(3):e52. 21821503 10.2196/jmir.1772 PMC3222162

[25] Sieverink F, Kelders SM, van Gemert-Pijnen JE. Clarifying the Concept of Adherence to eHealth Technology: systematic review on when usage becomes adherence. J Med Internet Res. 2017;19(12):e8578. 10.2196/jmir.8578 PMC5738543 29212630

[26] Nadal C, Sas C, Doherty G. Technology Acceptance in Mobile Health: scoping review of definitions, models, and measurement. J Med Internet Res. 2020;22(7):e17256. 32628122 10.2196/17256 PMC7381045

[27] Gronier G, Baudet A. Psychometric evaluation of the F-SUS: creation and validation of the French Version of the System Usability Scale. Int J Human–Computer Interact. 2021;37(16):1571–82.

[28] Brooke JSUS. A retrospective. J Usability Stud. 2023;8(2):29–40.

[29] Association AP. Diagnostic and Statistical Manual of Mental Disorders: Dsm-5. 5th Revised edition. Washington, D.C: American Psychiatric Publishing; 2013. 1 p.

[30] Lukasiewicz M, Fareng M, Benyamina A, Blecha L, Reynaud M, Falissard B. Ecological momentary assessment in addiction. Expert Rev Neurother. 2007;7(8):939–50. 17678488 10.1586/14737175.7.8.939

[31] Falcón E, McClung CA. A role for the circadian genes in drug addiction. Neuropharmacology. 2009;56(Suppl 1):91–6. 18644396 10.1016/j.neuropharm.2008.06.054 PMC2635341

[32] Goodman J, Packard MG. Memory Systems and the addicted brain. Front Psychiatry. 2016;7:24. 26941660 10.3389/fpsyt.2016.00024 PMC4766276

[33] Bora E, Zorlu N. Social cognition in alcohol use disorder: a meta-analysis. Addict Abingdon Engl. 2017;112(1):40–8. 10.1111/add.13486 27287050

[34] Bangor A, Kortum PT, Miller JT. An empirical evaluation of the System Usability Scale. Int J Hum-Comput Interact. 2008;24(6):574–94.

[35] McTavish FM, Chih MY, Shah D, Gustafson DH. How patients recovering from alcoholism use a smartphone intervention. J Dual Diagn. 2012;8(4):294–304. 23316127 10.1080/15504263.2012.723312 PMC3541672

[36] Klingemann J, Wieczorek Ł. Mobile application recovery support for patients with an alcohol use disorder. Acceptance, usability, and perceived helpfulness. J Addict Dis. 2022;40(4):559–67. 35274601 10.1080/10550887.2022.2049177

[37] Kelly PJ, Beck AK, Baker AL, Deane FP, Hides L, Manning V et al. Feasibility of a Mobile Health App for Routine Outcome Monitoring and Feedback in Mutual Support Groups Coordinated by SMART Recovery Australia: Protocol for a Pilot Study. JMIR Res Protoc [Internet]. 2020 Jul 9 [cited 2021 Mar 30];9(7). https://www.ncbi.nlm.nih.gov/pmc/articles/PMC7380906/10.2196/15113 PMC7380906 32673272

[38] Jakob R, Harperink S, Rudolf AM, Fleisch E, Haug S, Mair JL, et al. Factors influencing adherence to mHealth apps for Prevention or Management of Noncommunicable diseases: systematic review. J Med Internet Res. 2022;24(5):e35371. 35612886 10.2196/35371 PMC9178451

[39] Witkiewitz K, Desai SA, Bowen S, Leigh BC, Kirouac M, Larimer ME. Development and evaluation of a mobile intervention for heavy drinking and smoking among college students. Psychol Addict Behav. 2014;28(3):639–50. 25000269 10.1037/a0034747 PMC6143292

[40] Waselewski ME, Flickinger TE, Canan C, Harrington W, Franklin T, Otero KN, et al. A Mobile Health App to support patients receiving medication-assisted treatment for opioid use disorder: development and feasibility study. JMIR Form Res. 2021;5(2):e24561. 33620324 10.2196/24561 PMC7943342

[41] Malte CA, Dulin PL, Baer JS, Fortney JC, Danner AN, Lott AMK, et al. Usability and acceptability of a Mobile App for the self-management of Alcohol Misuse among veterans (step away): pilot cohort study. JMIR MHealth UHealth. 2021;9(4):e25927. 33830064 10.2196/25927 PMC8063094

[42] Hsu M, Martin B, Ahmed S, Torous J, Suzuki J, Smartphone, Ownership. Smartphone utilization, and Interest in Using Mental Health Apps to address Substance Use disorders: Literature Review and cross-sectional Survey Study Across two sites. JMIR Form Res. 2022;6(7):e38684. 35797102 10.2196/38684 PMC9305402

[43] Carreiro S, Newcomb M, Leach R, Ostrowski S, Boudreaux ED, Amante D. Current reporting of usability and impact of mHealth interventions for substance use disorder: a systematic review. Drug Alcohol Depend. 2020;215:108201. 32777691 10.1016/j.drugalcdep.2020.108201 PMC7502517

[44] Sardi L, Idri A, Fernández-Alemán JL. A systematic review of gamification in e-Health. J Biomed Inf. 2017;71:31–48. 10.1016/j.jbi.2017.05.011 28536062

[45] Tran S, Smith L, El-Den S, Carter S. The Use of Gamification and incentives in Mobile Health apps to improve Medication Adherence: scoping review. JMIR MHealth UHealth. 2022;10(2):e30671. 35188475 10.2196/30671 PMC8902658

[46] Torous J, Bucci S, Bell IH, Kessing LV, Faurholt-Jepsen M, Whelan P, et al. The growing field of digital psychiatry: current evidence and the future of apps, social media, chatbots, and virtual reality. World Psychiatry off J World Psychiatr Assoc WPA. 2021;20(3):318–35. 10.1002/wps.20883 PMC8429349 34505369

[47] Bhugra D, Tasman A, Pathare S, Priebe S, Smith S, Torous J, et al. The WPA-Lancet Psychiatry Commission on the Future of Psychiatry. Lancet Psychiatry. 2017;4(10):775–818. 28946952 10.1016/S2215-0366(17)30333-4

[48] Werntz A, Amado S, Jasman M, Ervin A, Rhodes JE. Providing human support for the Use of Digital Mental Health Interventions: systematic Meta-review. J Med Internet Res. 2023;25:e42864. 36745497 10.2196/42864 PMC9941905

[49] Krebs P, Duncan DT. Health App Use among US Mobile Phone Owners: a National Survey. JMIR MHealth UHealth. 2015;3(4):e101. 26537656 10.2196/mhealth.4924 PMC4704953

[50] Smith KA, Blease C, Faurholt-Jepsen M, Firth J, Van Daele T, Moreno C, et al. Digital mental health: challenges and next steps. BMJ Ment Health. 2023;26(1):e300670. 37197797 10.1136/bmjment-2023-300670 PMC10231442

