# Supplementary material for: Acceptability of “DIDE”, a mobile application designed at facilitating care adherence of patients with substance use disorder
Source: Addict Sci Clin Pract. 2024 Oct 15;19:72. doi: 10.1186/s13722-024-00500-7 (PMC11476181; doi:10.1186/s13722-024-00500-7)
Supplement: Supplementary file 1 — Supplementary Material 1 [file 13722_2024_500_MOESM1_ESM.docx]

**Additional file 1 : Application questionnaires**

**« Consumption diary » questionnaire:**

- Time and date (filled automatically on questionnaire’s launch).
- Cocaine (+/-).
- Cannabis (+/-).
- Alcohol (+/-).
- For each of these three products, users can increment or decrement the counter by one using the + and – buttons. User can modify this counter back and forth during the day. The number resets each day.
- “Would you like to discuss with your healthcare team?” (Yes / No). If the user answers “Yes”, they are redirected to the messaging system within the app.

**« Rhythm of life » questionnaire:**

- Time and date (filled automatically on questionnaire’s launch).
- “How much hours did you sleep last night?” (a tap on the screen opens a numeric keypad to fill the form).
- “At what time did you get up?” (a tap on the screen opens a clock).
- “How many meals did you have?” (a tap on the screen opens a numeric keypad to fill the form).
- “Would you like to consult your to-do list for the day?” (Yes / No). If the user answers “Yes”, they are redirected to their to-do list within the app.

**« Feeling » questionnaire:**

- Time and date (filled automatically on questionnaire’s launch).
- “Is everything all right?” (user can answer using a scale graduated from 1 to 10).

**« Craving » questionnaire:**

- Time and date (filled automatically on questionnaire’s launch).
- “Would you like to indicate the intensity of your craving?” (Yes / No). If the user answers “Yes”, a scale graduated from 1 to 10 appears.
- “Would you like to discuss it with your healthcare team?” (Yes / No). If the user answers “Yes”, they are redirected to the messaging system within the app.
- “Would you like to get recommendations of forums or remote emotional support systems?” (Yes / No). If the user answers “Yes”, they are redirected to a page listing user forums and emotional support systems.
